# A cost-effective ultrasound model for demonstrating the whirlpool sign in testicular torsion

**DOI:** 10.4102/jcmsa.v3i1.181

**Published:** 2025-04-16

**Authors:** Hanlie Dreyer, Izak Petrus Scholtz, Ahmed Adam, Abdullah E. Laher

**Affiliations:** 1 Department of Emergency Medicine, Faculty of Health Sciences, University of the Witwatersrand, Johannesburg, South Africa; 2 Department of Emergency Medicine, Faculty of Health Sciences, School of Clinical Medicine, University of the Witwatersrand, Johannesburg, South Africa; 3 Department of Urology, Faculty of Health Sciences, University of the Witwatersrand, Johannesburg, South Africa

**Keywords:** testicular torsion, ultrasound training, whirlpool sign, low-cost medical simulation, emergency medicine

## Abstract

Testicular torsion is a urological emergency requiring rapid diagnosis to prevent testicular loss, with the whirlpool sign on ultrasound being a critical indicator. In resource-limited settings, access to specialised ultrasound training is often constrained. To address this, we developed a cost-effective, reusable ultrasound training model that replicates the whirlpool sign using easily accessible materials. Our model consists of a boiled egg and coiled tubing set in gelatine, with iodine simulating blood flow. Emergency Medicine residents used the model during hands-on training sessions. This model offers a simple yet effective way to improve diagnostic skills in resource-constrained settings, potentially enhancing early detection and outcomes for testicular torsion.

## Introduction

Scrotal pathology is a frequent cause of Emergency Department visits, with testicular torsion representing a critical urological emergency that requires immediate diagnosis and intervention to prevent testicular loss. Torsion occurs when the testicle twists around the spermatic cord, obstructing blood flow and causing progressive ischaemia and, eventually, necrosis. Timely intervention, ideally within 6–8 h, is essential to preserve the testicle.^1,2^

Testicular torsion predominantly affects males aged 12–18 years old, with an incidence of 10% – 15% in the paediatric population.^3^ Although precise data for sub-Saharan Africa is limited, incidence rates are considered comparable to developed countries.^4^ The initial step in managing a suspected testicular torsion is to have a high index of suspicion, rapidly perform confirmatory tests, and then immediately refer these patients to a urologist for urgent surgical exploration and detorsion.^2^

Ultrasonography is considered the gold standard modality for the diagnosis of testicular torsion. It facilitates the evaluation of testicular size, echotexture, the presence of fluid collections, as well as abnormalities on Doppler imaging.^5^ When performed by experienced providers, it demonstrates a diagnostic sensitivity of 82% and a specificity of 100% in diagnosing testicular torsion.^6^

A sonographic finding known as the ‘whirlpool sign’, indicating a twisted spermatic cord, is pathognomonic for the diagnosis of testicular torsion, with a reported sensitivity of 92% and specificity of 99% in adults and paediatric populations. The sign demonstrates the spiral-like patterns of the twisted spermatic cord.^3^

In resource-limited settings, access to specialised ultrasound training often remains a significant challenge.^7^ To bridge this gap, we have developed a cost-effective ultrasound model that demonstrates the testicular whirlpool sign and may improve the diagnostic skills of clinicians.

## Materials and model construction

The phantom model was created using inexpensive and readily available materials, making it suitable for resource-constrained settings. The model is constructed to simulate the appearance of a twisted spermatic cord on ultrasound, using a boiled egg to represent the testicle and a short piece of tubing to mimic the twisted cord. Gelatine is utilised to simulate soft tissue, while iodine is used to mimic blood circulation.

### Materials

- 50 g gelatine powder
- 30 mL 70% alcohol
- 500 mL water
- Boiled egg × 1
- Plastic microwave-safe container
- 100 cm length of tubing (e.g. IV tubing; length dependent on how many loops are made)
- Food colouring solution: 5 mL food colouring to be diluted in water. 250 mL of the coloured water for injection into the tubing. (You can adjust the amount of colouring to change the appearance of the water)
- Ultrasound device with a high-frequency probe. Testicular or small parts preset option for probe configuration
- Ultrasound gel

### Model construction

1. Prepare a gelatine mixture by combining 50 g of gelatine powder with 250 mL of water and 30 mL of 70% alcohol. Mix thoroughly and allow to cool for 30 min, skimming off any surface foam. Pour half the mixture into the container and refrigerate.
2. Attach the tubing to the posterior aspect of the boiled egg, twisting it to simulate the twisted spermatic cord.
3. Once the initial gelatine layer has set, position the egg with tubing within the bowl, then cover the rest of the model with the remaining gelatine mixture to encase the model fully. Refrigerate until set.
4. To simulate the whirlpool effect, inject the food colouring solution into the tubing, while the operator performs the ultrasound study by placing the linear probe over the twisted tubing.

## Cost analysis

This model was designed with cost-effectiveness as a primary goal to ensure accessibility in resource-limited settings. The breakdown of material costs is as follows:

- Gelatine powder: R40.66 ($2.20)
- Seventy per cent alcohol: R11.09 ($0.60)
- Boiled egg: R3.70 ($0.20)
- Food colouring: R20.33 ($1.10)
- Plastic microwave-safe container: R16.63 ($0.90)
- Tubing: R25.88 ($1.40)

Total cost: ZAR 118.29 ($6.40; $1.00 = R 18.48).

## Implementation and training application

**FIGURE 1:**
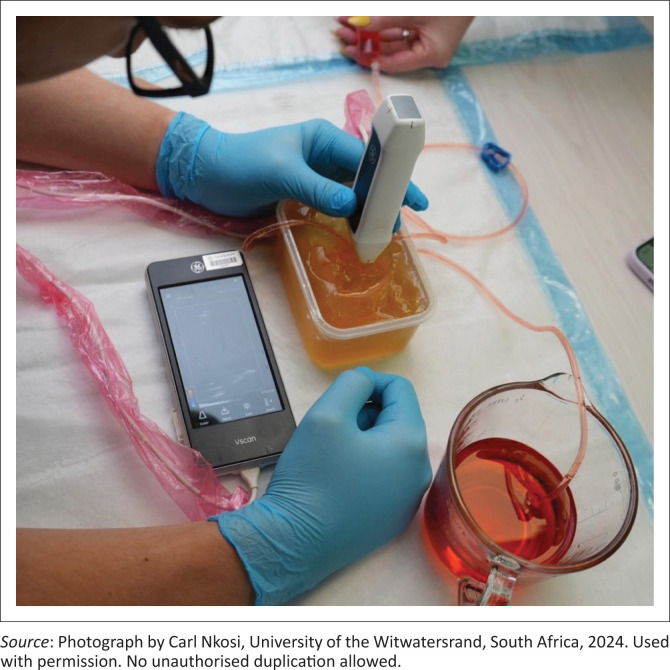
Ultrasound trainer model for demonstrating the whirlpool sign.

**FIGURE 2:**
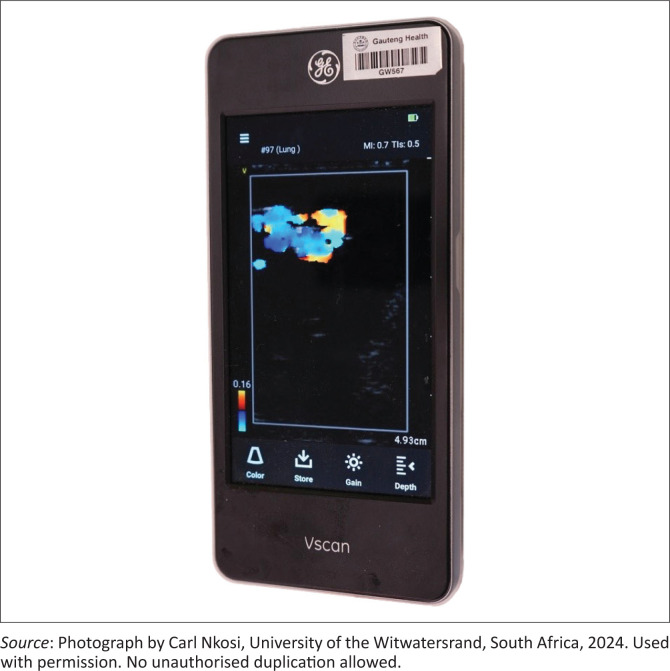
Ultrasound generated image of torsed spermatic cord with the whirlpool sign.

To maximise the phantom’s impact, we integrated it into a structured emergency medicine training session. All registrars received a lecture on the fundamentals of testicular ultrasound, including an explanation of the whirlpool signs and its sensitivity and specificity for testicular torsion. Each participant utilised a handheld ultrasound device on the phantom (see Figure 1). The diagnostic scenario was depicted, allowing the participant to observe the whirlpool sign on the phantom (see Figure 2). The model was not formally assessed within a research study.

This cost-effective, reusable phantom presents a valuable training tool for Emergency Medicine, Urology and Radiology curricula, where simulation of high-stakes, time-sensitive conditions is critical. Standardising the use of this phantom across training sessions ensures consistent practice and familiarity with diagnostic signs, helping trainees build confidence in recognising and diagnosing testicular torsion under simulated emergency conditions.

Regular skill assessments can further reinforce proficiency. Future research could focus on quantifying improvements in diagnostic accuracy and time efficiency among trainees who practice with this model, providing data on its educational impact.

## Conclusion

This low-cost ultrasound phantom model offers a practical, accessible method for teaching the recognition of the whirlpool sign, a crucial ultrasound feature for diagnosing testicular torsion. Its design allows Emergency Medicine registrars to gain hands-on experience in a safe, simulated environment, fostering confidence and diagnostic precision. Given the urgency of timely intervention in testicular torsion, early and accurate recognition of the whirlpool sign is essential to prevent testicular loss. This model serves as an effective educational tool, particularly in resource-constrained settings, by providing trainees with critical skills that enhance patient care in emergencies.
